# Supplementary material for: DNA-Binding Activity of CAMTA3 Is Essential for Its Function: Identification of Critical Amino Acids for Its Transcriptional Activity
Source: Cells. 2023 Aug 2;12(15):1986. doi: 10.3390/cells12151986 (PMC10417383; doi:10.3390/cells12151986)
Supplement: Supplementary file 1 [file cells-12-01986-s001.zip › supplementary figures.pdf]

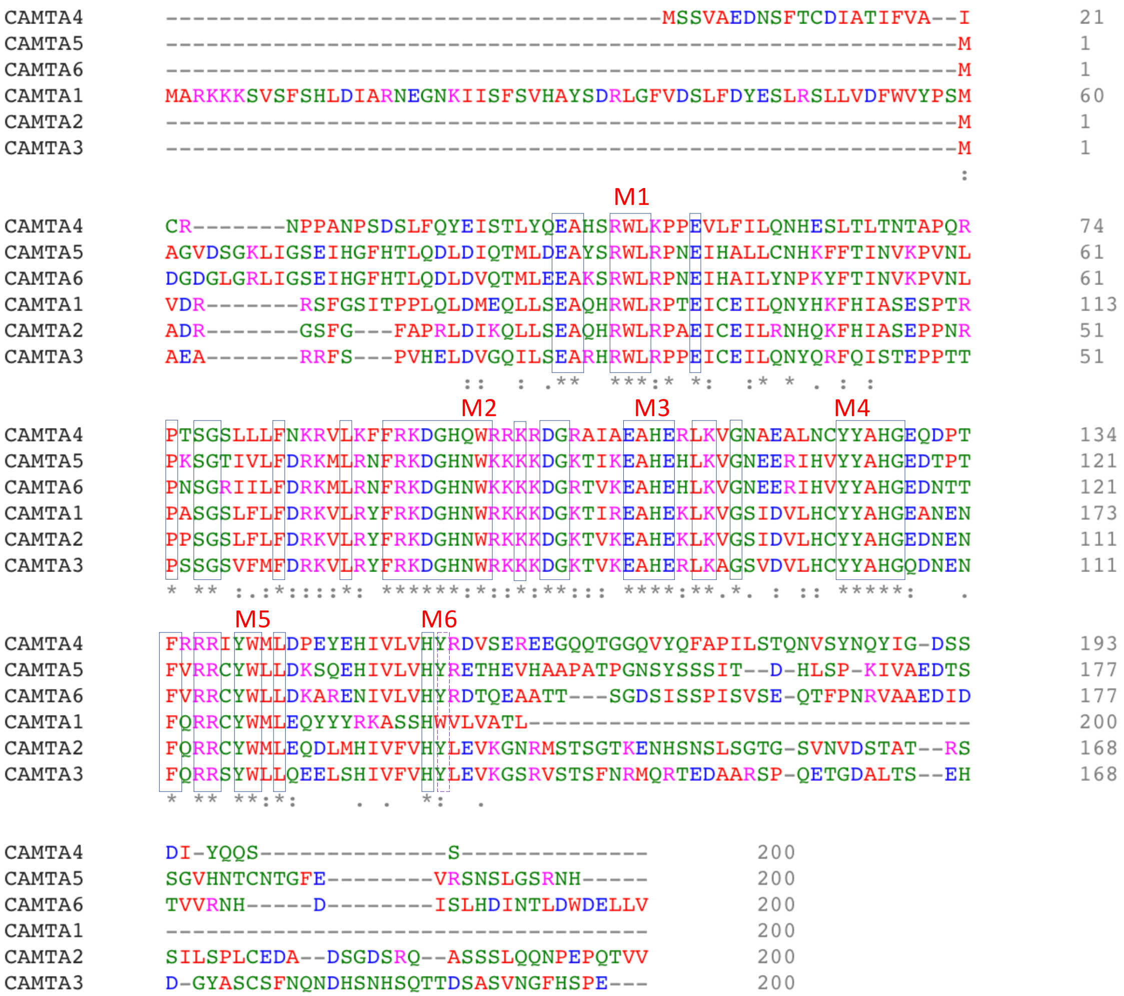

Supplementary Figure S1. Identification of evolutionarily conserved amino acid residues in the CG-1 domain of six CAMTAs in *Arabidopsis thaliana*. Clustal W alignment of first 200 amino acids (encompassing NLS and CG-1 domain) sequence in the CG-1 domain of AtCAMTAs 1-6. The stretches of highly conserved amino acid residues in all six CAMTAs proteins are marked by black boxes. Based upon the sequence logo in Fig. 1, the corresponding highly conserved amino acids with >4-bit score are indicated by letters M1-M6 on top of the amino acid.

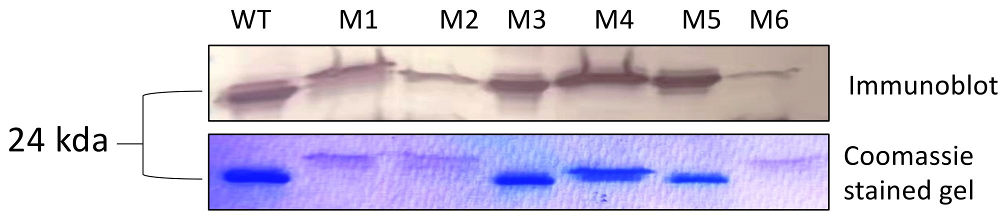

Supplementary figure S2. Expression and purification of WT and CG-1 mutants in *E. coli*. Immunoblot analysis of His-column purified protein of WT and CG-1 mutants (M1 to M6) using anti-His antibody.

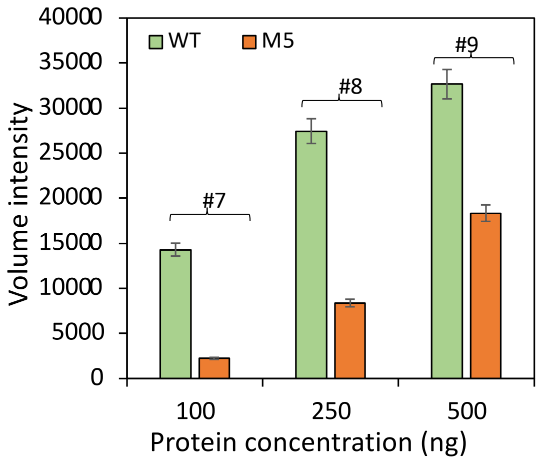

Supplementary figure S3. Significant differences in the binding activity of WT and M5 CG-1 mutant to the DNA probe. Comparison of the binding capacity of WT and M5 mutant to *PDF1.4* probe. Based on Fig. 2b and 3b, the signal volume intensity of bound DNA-Protein complex and unbound (DNA probe only) is calculated for each lane at a different protein concentration using image analysis software (Image Lab 6.0.1 version, Bio-Rad) provided by the manufacturer. Lane numbers are indicated on top of the bars.

RSRE:NOS

AGTTACATAACGCGTTTTTAGATATCATAACGCGTTTTTAGGATCCATAACGCGTTTTTATCTAGAATAACGCGTTTTTACTTGCGCGTTCAAAAG
TCGCCTAAGGTCACTATCAGCTAGCAAATATTTCTTGTCAAAAATGCTCCACTGACGTTCCATAAATTCCCCTCGGTATCCAATTAGAGT

mRSRE:NOS

CTATGTTCACCACTGATTATGAGAAGGTTAGCCTCTTCAATTTCAGAAAGAATGCTGATCCACAGATGGTTAGAGAGGCTAGAATAACATGCTT
TTACTTGCGCGTTCAAAAGTCGCCTAAGGTCACTATCAGCTAGCAAATATTTCTTGTCAAAAATGCTCCACTGACGTTCCATAAATTCCCCTCGG
TATCCAATTAGAGT

Supplementary figure S4. Generation of *RSRE::NOS and mRSRE::NOS* promoter for the reporter construct.
The sequence of *RSRE* [*CGCGTT*] elements (highlighted in green) included in the upstream region of the *NOS* minimal promoter (highlighted in grey) interspersed by other bases was used for the synthesis of *RSRE::NOS* promoter that was cloned upstream to the luciferase gene. For the synthesis of the mutant version *mRSRE::NOS* promoter, the *RSRE* elements (highlighted as magenta) and surrounding sequences were altered keeping the *NOS* minimal promoter (highlighted as grey) intact.

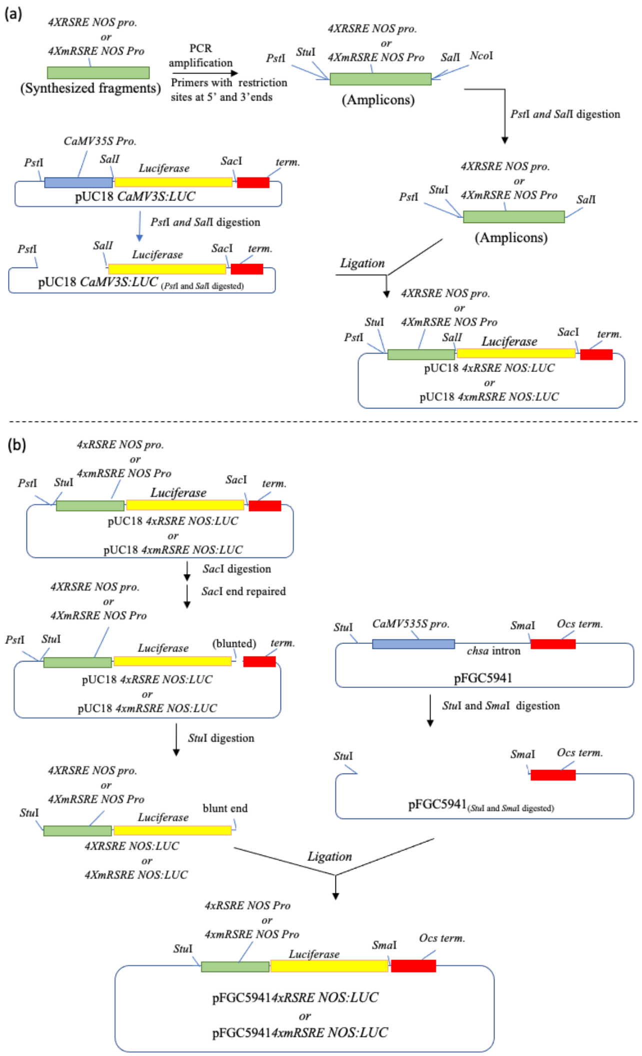

Supplementary figure S5. Schematic diagram illustrating the strategy used to generate *RSRE::NOS:LUC* and *mRSRE::NOS: LUC* constructs for *Agrobacterium*-mediated transient expression analysis.
