# Supplementary material for: DNA-Binding Activity of CAMTA3 Is Essential for Its Function: Identification of Critical Amino Acids for Its Transcriptional Activity
Source: Cells. 2023 Aug 2;12(15):1986. doi: 10.3390/cells12151986 (PMC10417383; doi:10.3390/cells12151986)
Supplement: Supplementary file 1 [file cells-12-01986-s001.zip › Table S1.pdf]

Table S1. Primers used for cloning, genotyping, RT-PCR and generation of mutants.

1	AtSR1/CAMTA3-FW	5'- CCA TTT AAA TAT GGC GGA AGC AAG ACG ATT CAG CCC A-3'
2	AtSR1/CAMTA3-RW	5'- CGC GGA TCC TTA ACT GGT CCA CAA AGA TGA GGA CAT A-3'
3	Q5SDM_76/77(W/A)-FW	5’-ACGACATCGAGCGCTTCGTCCTCC-3'
4	Q5SDM_76/77(W/A)-RW	5’-GCTTCTGAGAGTATTTGTC-3'
5	Q5SDM_223/234(W/A)-FW	5'-CGGTCACAATGCGAGGAAGAAAAAAGATGG-3'
6	Q5SDM_223/234(W/A)-RW	5'-TCTTTCCTGAAGTATCTGAG-3'
7	Q5SDM_262/263(H/A)-FW	5'-GAAGGAAGCTGCTGAGAGGTTGAAGGC-3'
8	Q5SDM_262/263(H/A)-RW	5'-ACAGTCTTTCCATCTTTTTTC-3'
9	Q5SDM_304/307(YY/AA)-FW	5'TCTACATTGTGCCGCTGCGCATGGACAGGACAATGAAAACTTTC-3'
10	Q5SDM_304/307(YY/AA)-RW	5'-ACATCAACGCTTCCCGCC-3'
11	Q5SDM_352/353(W/A)-FW	5'-ACGCAGTTACGCGTTGCTTCAGGAAG-3'
12	Q5SDM_352/353(W/A)-RW	5'-CTTTGAAAGTTTTCATTGTCCTG-3'
13	Q5SDM_394/395(Y/A)-FW	5'-TTTTGTTCACGCCCTCGAAGTTAAGG-3'
14	Q5SDM_394/395(Y/A)-RW	5'-ACTATGTGGGAAAGTTCTTC-3'
15	BamHI-SR1-DBD-FW	5’-CGCGGATCCGATGGCGGAAGCAAGACGATTC-3’
16	XhoI-SR1-DBD-RW	5’-CCGCTCGAGCGCGTCTTCAGTCCTTTGCATC-3’
17	XhoI-SR1-FL-RW	5’-CCGCTCGAGACTGGTCCACAAAGATGAGGAC-3’
18	StuI-RSRE	5’-AAAACTGCAGATAGAAGGCCTA-3’
19	StuI-mRSRE	5’-AAAACTGCAGATAGAAGGCCTA-3’
20	RSRE NOSCH-FW	5’-AAAACTGCAGATAGAAGGCCT-3’
21	RSRE NOSCH-RW	5’-CATGCCATGGCATGGTCGACA-3’
22	PDF1.4 PRO	5’-CGTGAAAGTCACGCGCCAAGAACAAA-3’Biotin
23	PDF1.4 PRO -COMP	5’-TTTGTTCTTGGCGCGTGACTTTCACG-3’
23	Q-SR1-FW	5’CTCGGGAGGAGACTGAAATTG-3’
24	Q-SR1-RW	5’AGGAGCAACACATTGGAGAATA-3’
25	EDS1-FW	5’-TCGAAGGGGACATAGATTGGATC-3’
26	EDS1-RW	5’-ATGTACGGCCCTGTGTCTTCGTTC-3’
27	ICS1-FW	5’-TATCTCCGGCAGCCGCCACT-3’
28	ICS1-RW	5’-ACGCCGGAGGAAAACGACGG-3’
29	CBP60G-FW	5’-TCGAAGCTGAGGATGGTTCT-3’
30	CBP60G-RW	5’TAAATCCCTCAACGGTCCAG-3’
31	ALPHA-DOX1-AT3G01420-FW	5’-ACGTCGACTTAGCTGCTTTAG-3’
32	ALPHA-DOX1-AT3G01420-RW	5’-CTCCGTTAGATCTTCCCACTTG-3’
33	COR15-AT2G42540-FW	5’-CTCAGTTCGTCGTCGTTTCT-3’
34	COR15-AT2G42540-RW	5’-GTTGAGGTCATCGAGGATGTT-3’
35	KIN1-AT5G15960-FW	5’GCTGAGGAGAAGAGCAATGT-3’
36	KIN1-T5G15960-RW	5’CCGCATCCGATACACTCTTT-3’
37	ACTIN2-FW	5’-GGCAAGTCATCACGATTGG-3’
38	ACTIN2-RW	5’-CAGCTTCCATTCCCACAAAC-3’
39	R3-SR1 FW	5'-TTGGCGCGCCATGGGACTTACATCTGATCGTACC-3'
40	R3-SR1 RW	5'-CGGGATCCTTAAATACCATTAGATATAACATCT-3'
41	R4-SR1 FW	5’-TTGGCGCGCCATGATTCTCCAATGTGTTGCTCCT-3’
42	R4-SR1 RV	5’-CGGGATCCTTAATCAACGCTTACACCAGCGAT-3’
